# Integrated Analysis of Clinical and Microbiome Risk Factors Associated with the Development of Oral Candidiasis during Cancer Chemotherapy

**DOI:** 10.3390/jof5020049

**Published:** 2019-06-13

**Authors:** Patricia I. Diaz, Bo-Young Hong, Amanda K. Dupuy, Linda Choquette, Angela Thompson, Andrew L. Salner, Peter K. Schauer, Upendra Hegde, Joseph A. Burleson, Linda D. Strausbaugh, Douglas E. Peterson, Anna Dongari-Bagtzoglou

**Affiliations:** 1 Department of Oral Health and Diagnostic Sciences, School of Dental Medicine, UConn Health, Farmington, CT 06030, USA; Bo-young.Hong@jax.org (B.-Y.H.); linda.choquette@jax.org (L.C.); Anthompson@uchc.edu (A.T.); peterson@nso.uchc.edu (D.E.P.); 2 The Jackson Laboratory for Genomic Medicine, Farmington, CT 06032, USA; 3 Department of Molecular and Cell Biology, University of Connecticut, Storrs, CT 06269, USA; amanda.dupuy@uconn.edu (A.K.D.); linda.strausbaugh@uconn.edu (L.D.S.); 4 Department of Medical Oncology, Hartford Healthcare, Hartford, CT 06106, USA; Andrew.Salner@hhchealth.org (A.L.S.); Peter.Schauer@hhchealth.org (P.K.S.); 5 Department of Medicine, UConn Health, Farmington, CT 06030, USA; uhegde@uchc.edu; 6 Department of Community Medicine and Health Care, UConn Health, Farmington, CT 06032, USA; burleson@uchc.edu

**Keywords:** oral candidiasis, microbiome, cancer chemotherapy, risk factors

## Abstract

Oral candidiasis is a common side effect of cancer chemotherapy. To better understand predisposing factors, we followed forty-five subjects who received 5-fluorouracil- or doxorubicin-based treatment, during one chemotherapy cycle. Subjects were evaluated at baseline, prior to the first infusion, and at three additional visits within a two-week window. We assessed the demographic, medical and oral health parameters, neutrophil surveillance, and characterized the salivary bacteriome and mycobiome communities through amplicon high throughput sequencing. Twenty percent of all subjects developed oral candidiasis. Using multivariate statistics, we identified smoking, amount of dental plaque, low bacteriome and mycobiome alpha-diversity, and the proportions of specific bacterial and fungal taxa as baseline predictors of oral candidiasis development during the treatment cycle. All subjects who developed oral candidiasis had baseline microbiome communities dominated by *Candida* and enriched in aciduric bacteria. Longitudinally, oral candidiasis was associated with a decrease in salivary flow prior to lesion development, and occurred simultaneously or before oral mucositis. Candidiasis was also longitudinally associated with a decrease in peripheral neutrophils but increased the neutrophil killing capacity of *Candida albicans*. Oral candidiasis was not found to be associated with mycobiome structure shifts during the cycle but was the result of an increase in *Candida* load, with *C. albicans* and *Candida dubliniensis* being the most abundant species comprising the salivary mycobiome of the affected subjects. In conclusion, we identified a set of clinical and microbiome baseline factors associated with susceptibility to oral candidiasis, which might be useful tools in identifying at risk individuals, prior to chemotherapy.

## 1. Introduction

Cancer chemotherapy is associated with comorbidities, with the oral cavity representing one of the most severely affected body sites. Oral complications include mucositis, which presents as erythema or ulceration of the non-keratinized mucosa, and oral candidiasis, which commonly presents as white, detachable, pseudomembranous mucosal lesions [1,2,3]. Oral candidiasis incidence is 20–40% in chemotherapy recipients, depending on dose, duration, and regimen type [3]. In patients with hematologic malignancies who are treated aggressively with a high dose of cytotoxic chemotherapy, the incidence of oral candidiasis is higher and the consequences from this infection are more severe than in patients receiving low-dose chemotherapeutic regimens. In addition to burning pain and taste changes that compromise nutrition, a serious consequence of oral candidiasis in that the population is an increased risk to lethal, disseminated infection [4,5,6,7]. A previous large-scale retrospective study of hematologic cancer patients identified more intensive chemotherapy and longer lasting severe leukopenia as clinical parameters increasing the risk of oral and systemic candidiasis. Importantly, in this large cohort, almost all patients who developed oral candidiasis later developed fatal, systemic candidiasis [7]. Despite important advances in managing chemotherapy patients with oral and systemic fungal infections over the past three decades, these infections remain a significant clinical problem in this population [8,9]. Thus, the identification of risk factors for oral infection is of paramount importance in patients undergoing chemotherapy. Prospective, longitudinal studies with newer cancer chemotherapy regimens are needed to identify the underlying factors of increased susceptibility to oral candidiasis. 

Resistance to opportunistic infections in the oral cavity is the result of a balanced interaction between a complex oral microbiome and the host’s protective defenses. The oral microbiome is a diverse community with hundreds of bacterial and fungal taxa [10,11,12]. These communities live in constant cross-talk with the adjacent oral mucosa, eliciting the production of antimicrobial peptides and recruitment of leukocytes that contribute to the maintenance of the microbiota, in a commensal state [13,14,15]. Since not all patients subjected to a similar chemotherapy regimen develop oral candidiasis, it is likely that inter-subject variability in the oral microbiome is related to susceptibility, with specific fungal and bacterial microbiome profiles setting the stage for infection. 

Chemotherapy is also associated with myelosuppression, with severe long-lasting neutropenia or alterations in neutrophil chemotaxis placing patients at risk of systemic candidiasis [16,17]. In vitro assays suggest that chemotherapeutics affect neutrophil function [18]. Neutrophils offer protection from the oral overgrowth of *Candida albicans* [19]—the fungus most frequently associated with oral candidiasis [20]. Thus, decreased neutrophil oral availability and alterations in neutrophil function as a consequence of chemotherapy, combined with mucosal injury might affect oral candidiasis risk. Moreover, a decrease in salivary flow rate (SFR), reported to occur during chemotherapy [21], could lead to an impaired delivery of candidacidal antimicrobials [22], predisposing to oral candidiasis. Other chemotherapy-associated treatments such as antibiotics, could affect the oral microbiome predisposing to *Candida* overgrowth. At non-oral mucosal barriers, antibiotic perturbation of resident bacteria has been linked to candidiasis [23], but it is less clear if antibiotic intake confers a risk in the oral cavity. Instead, *C. albicans* and certain oral commensal bacteria have been suggested to have a synergistic relationship [24,25] and a recent mouse model of chemotherapy-associated candidiasis has provided direct evidence to support this concept [26]. Delineation of factors that predispose individuals undergoing chemotherapy to oral candidiasis, could lead to clinical tools for the application of preventive approaches, based on individual risk.

Accordingly, this study investigated the factors associated with the development of oral candidiasis during chemotherapy. Forty-five patients undergoing 5-fluorouracil (5-FU)- or doxorubicin-based chemotherapy were evaluated at 4 time-points during one chemotherapy cycle, including a baseline visit prior to the first infusion and three subsequent visits within a two week window. The variables evaluated included the demographic, medical, and oral clinical characteristics of subjects, together with an assessment of neutrophil surveillance, bacterial and *Candida* burdens, and the oral bacteriome and mycobiome. Our goal was to identify patient characteristics related to oral candidiasis susceptibility in this clinical setting.

## 2. Materials and Methods

### 2.1. Study Design

This observational prospective study was approved by the Institutional Review Board at UConn Health (IRB number IE-11-037J-2) and conformed with the rules of the Declaration of Helsinki of 1975 (https://www.wma.net/what-we-do/medical-ethics/declaration-of-helsinki/), as revised in 2013, and with STROBE guidelines. Subjects treated for a solid tumor—with either a 5-fluorouracil (5-FU)- or a doxorubicin (Adriamycin)-based chemotherapy regimen—were recruited at the Neag Comprehensive Cancer Center of UConn Health and at the Helen and Harry Gray Cancer Center at Hartford Hospital. Subjects were seen at four visits, which included a baseline visit completed prior to subjects commencing chemotherapy. The day of the first infusion was considered day 0, after which subjects were evaluated at three additional visits at days +3 (±2), +9 (±2), and +14 (±2). A detailed characterization of this study cohort, with respect to the development of oral mucositis, has been previously published by our group [27]. Our previous report included 49 subjects. In the current study, 45 subjects were included in the analyses as 4 subjects did not have complete clinical oral evaluations to assess oral candidiasis at all visits.

### 2.2. Demographic and Medical Data Collection and Oral Evaluation

Medical information was obtained from questionnaires and medical charts. All subjects received an oral evaluation at baseline, including an assessment of the presence of periodontitis, via the Community Periodontal Index of Treatment Needs (CPITN) [28], presence and type of prosthetic restorations, number of teeth, and presence of visible cavitated carious lesions. In addition, the following data were collected at all visits—presence of oral candidiasis defined as detachable pseudomembranous white lesions on the oral mucosa; unstimulated SFR; and amount of plaque on teeth, as determined via the Plaque Index of Silness and Löe [29].

### 2.3. Saliva Collection

At each visit, unstimulated saliva was collected to determine SFR and for microbiome evaluation. Participants were instructed to avoid eating or drinking anything other than water, for one hour prior to each study visit. Unstimulated saliva was collected for 5 min by having subjects lean forward over a sterile funnel attached to a vial placed in ice. Saliva was weighted to determine the salivary flow rate, and then aliquoted and centrifuged at 2200 *g* for 10 min. Supernatants were removed and pellets were stored at −80 °C. 

### 2.4. Evaluation of Peripheral Blood and Oral Neutrophil Counts

Total counts and percentages of neutrophils were obtained from the complete blood counts performed by the hospital laboratory, using an automated Beckman Coulter analyzer. Oral cells were collected at all study visits by asking subjects to rinse with 10 mL of a bicarbonate solution for 30 s. Oral rinse samples were centrifuged at 1258× *g* for 15 min and the pellets were resuspended in Hanks’ Balanced Salt solution, supplemented with 2 μg/mL acridine orange. Granulocytes, characteristically stained as multilobulated cells with this nuclear stain, were counted in a hemacytometer, under a fluorescence microscope [30].

### 2.5. Evaluation of Peripheral Neutrophil Killing of C. albicans

At baseline and visit 4, 50 mL of blood were collected from a convenience subset of patients (n = 14) and neutrophils were isolated from the anticoagulated blood by dextran T-500 sedimentation, followed by density gradient centrifugation and erythrocytic lysis, through hypotonic shock. Cells were washed with Hank’s Balanced Salts Solution without calcium and magnesium, and were re-suspended in RPMI 1640 with 10 mM HEPES (Mediatech, Inc.). The resulting cell preparations consisted of more than 95% neutrophils, as assessed by the Wright–Giemsa stain and more than 98% were viable by trypan blue exclusion. The antimicrobial effector function of these cells was tested against *C. albicans* SC5314. Neutrophils were added to *C. albicans* at effector:target ratios ranging from 5:1 to 1:1. After incubation at 37 °C and 5% CO_2_ for 2 h, media were aspirated and neutrophils were lysed with sterile water. This was followed by quantification of metabolically active *C. albicans* via the XTT assay. Antifungal activity was calculated according to the following formula: % fungal damage = (1 − x/n) * 100, where x is the OD_450_ absorbance of experimental wells (*C. albicans* with effectors) and n is the absorbance of the control wells (*C. albicans* only) [31].

### 2.6. DNA Extraction and Generation of 16S rRNA Gene and ITS-1 DNA Libraries

DNA was extracted from saliva samples, separately for bacterial and fungal microbiome evaluation. For bacteria, we followed a previously described procedure using lysozyme and proteinase K treatment and the DNeasy Blood and Tissue kit (Qiagen) [32]. For fungi, the DNA extraction protocol involved bead beating with a matrix containing Lysing Matrix B (MP Biomedicals) and a very high density 0.5 mm yttrium-stabilized zirconium oxide (95% ZrO_2_ + 5% Y_2_O_3_) grinding media (YSZ) (Glen Mills Inc, Clifton, NJ, USA), followed by extraction using the FastDNA SPIN KIT (MP Biomedicals), as previously described [12]. Amplicon libraries of the 16S rRNA gene V1–V2 hypervariable regions were generated in triplicates, using fusion primers, which included universal primers 8F AGAGTTTGATCMTGGCTCAG or 361R CYIACTGCTGCCTCCCGTAG [33]. PCR conditions have been previously described [32]. For mycobiome characterization, fusion primers containing fungal-specific ITS1F forward primer [34] (CTTGGTCATTTAGAGGAAGTAA) or ITS2 reverse primer [35] (GCTGCGTTCTTCATCGATGC) were used to amplify the ITS1 sequences, in triplicates, as previously described [12]. The combined libraries were further purified and sequenced using 454 Titanium chemistry and the 454-GS-FLX sequencing platform (454 Life Sciences, Branford, CT).

### 2.7. Processing of Amplicon Sequences and Taxonomic Classification

16S rRNA gene reads were processed in mothur [36]. Primers and barcodes were trimmed, followed by the removal of sequences shorter than 200 bp, with homopolymers greater than eight nucleotides or ambiguous base calls. Sequences were then filtered using a 50 bp sliding window approach and an average quality score threshold of 35 [37]. Chimeric sequences were removed with UChime [38], in mothur. Sequences were then classified to the species level, by using the classify.seqs command and the Human Oral Microbiome database (HOMD) V14.5 as reference. The parameters used were—method = knn, search = blast, gapopen = −5, gapextend = −5, match = 4, mismatch = −5, and numwanted = 1, following recommendations by Al-Hebshi et al. [39]. This taxonomy assignment algorithm was validated by classifying the HOMD reference sequences, trimmed to include only the V1–V2 region, against the HOMD full length reference sequence database. With a few exceptions, all V1–V2 short sequences were correctly classified in the validation test. However, the following species could not be discriminated from each other—*Lactobacillus casei* and *Lactobacillus rhamnosus*; *Veillonella parvula* and *Veillonella dispar; Streptococcus mitis, Streptococcus pneumoniae* and *Streptococcus* sp. HOT423; and *Neisseria flavescens* and *Neisseria subflava*. Counts for species that could not be correctly identified were aggregated.

ITS-1 reads were also processed in mothur [36]. Sequences were trimmed, quality filtered, and chimeras removed, as described for 16S rRNA sequences. Sequences were then classified to genus level by using the classify.seqs command and a modified version of the Findley et al. database [40] as a reference, employing the recommended parameters (https://www.mothur.org/wiki/Findley_ITS_Database). Prior to this, a curation of the Findley et al. database was conducted to join the synonym taxa under one preferred name [20]. To accomplish this, reference sequences from synonym taxa were compared via BLAST against NCBI’s nucleotide type strain database and against the Fungal Metagenomics Project database. After confirming their genus identity, *Gueomyces* sequences were included under *Trichosporon*; *Lewia* under *Alternaria*; *Valsa* under *Cytospora*; *Coprinellus* and *Coprinopsis* under *Coprinus*; *Erythrobasidium* under *Rhodotorula*; *Cochliobolus* under *Curvularia*; *Filobasidium*, *Cystofilobasidium* and *Dioszegia* under *Cryptococcus*; and *Emericella* under *Aspergillus*. In addition, sequences of *Cladosporium*, *Toxicocladosporium*, *Aureobasidium*, *Kabatiella*, *Scleroconidioma* and *Candida* were added as they were underrepresented in the database. *Malassezia* and *Candida* ITS-1 sequences were further classified to the species level, using curated and aligned reference libraries and the software package pplacer [41], as previously described [40]. All sequences are available at the Short Reads Archive (Accession number PRJNA399163).

### 2.8. Alpha-Diversity Estimates for 16S rRNA gene and ITS-1 Amplicon Data

16S rRNA gene libraries were subsampled at 3074 reads and ITS-1 at 1338 reads, a threshold which was shown to yield sufficient depth, maximizing the number of samples included in the analysis [27]. Community alpha-diversity was evaluated via the non-parametric Shannon Index, as calculated in mothur. These metrics were constructed, based on the species-level taxonomic units for 16S rRNA gene reads and the genus-level for ITS-1 (with the exception of *Malassezia* and *Candida* that were speciated).

### 2.9. Evaluation of Salivary Bacterial and Candida Load via Real Time PCR

Total bacterial load in the saliva was measured via real-time PCR, using a TaqMan® probe and universal 16S rRNA gene primers, as previously described [42]. A standard curve, generated with serially-diluted genomic DNA from *Fusobacterium nucleatum* was used a reference. For quantification of the *Candida* load, we used *Candida*-specific ITS primers and SYBR Green (Roche) detection, as described [43]. A standard curve generated with genomic DNA obtained from serially diluted *C. albicans* SC5314 cells was used as a reference.

### 2.10. Statistical Analyses

Baseline nominal data were compared between the oral candidiasis-positive and negative subjects by Fisher’s exact test, and categorical data were compared via chi-square. Continuous data were evaluated via independent sample t-tests or Mann–Whitney U tests, according to the data distribution. The distribution of continuous data was tested for normality, using measures of Skewness and Kurtosis and the Shapiro–Wilk test in SPSS. Differences in the intake of medications during the cycle, according to the oral candidiasis incidence, were evaluated via Fisher’s exact tests. 

To evaluate the discriminant, demographic, clinical, or microbial baseline characteristics that could classify subjects that developed oral candidiasis during the cycle and the unaffected individuals, we performed sparse Partial Least Squares discriminant analysis (sPLS-DA) using the R package MixOmics [44]. Prior to this analysis, microbiome relative abundance data were filtered to exclude taxa present in less than 10% of baseline samples and data were transformed using a Centered Log Ratio transformation procedure [45]. The classification performance of the PLS-DA model was assessed with the perf function, using five-fold cross-validation, repeated ten times. Differences in the individual baseline proportions of identified discriminant taxa between affected and unaffected subjects, were also evaluated via Mann–Whitney Rank tests.

Longitudinal data were evaluated in subject-matched samples. Differences in subject-matched longitudinal data were evaluated via paired Wilcoxon Rank tests. We were also interested in the longitudinal changes in the presence of oral candidiasis signs and the relationship of these changes with other variables. We, therefore, modelled the change in each longitudinal variable across four visits, using linear or quadratic orthogonal polynomial contrasts. For this, each data point was multiplied by a standard coefficient (for a linear or quadratic four level contrast) and data were aggregated by subject. Correlations between linear or quadratic longitudinal change and other variables were determined via Spearman Rank tests and the significance threshold was adjusted using the Benjamini Hochberg false discovery rate method. Evaluation of the longitudinal covariation in microbial abundances and clinical parameters measured at the four visits of the study was performed using MixOmics. For this analysis, we used the multilevel function and compared microbial and clinical variables, using sparse Partial Least Squares (sPLS) regression. This multilevel multivariate approach takes into account the repeated measures within subjects and the use of sPLS reveals covariation patterns in all variables in an unsupervised manner.

## 3. Results

### 3.1. Evaluation of Baseline Characteristics Associated with Oral Candidiasis Incidence during a Chemotherapy Cycle

The characteristics of the subjects included in this study are summarized in Table 1 and Table 2. Of the 45 subjects receiving chemotherapy, 9 (20%) developed oral candidiasis during the cycle (Table 1). Our first goal was to identify factors at baseline, related to the development of oral candidiasis. Table 1 compares the demographic, medical, and oral clinical characteristics of patients at baseline, between subjects that developed oral candidiasis and those that remained candidiasis-free. No demographic variable was associated with oral candidiasis. Among the general characteristics evaluated, only smoking showed an association with oral candidiasis, with a greater percentage of current smokers and subjects with a smoking history in the candidiasis-positive group. Baseline salivary flow rate, which might be important for oral mucosal defenses [46], did not differ between groups.

Since neutrophil surveillance has been linked to the control of *C. albicans* growth and dissemination [19,47], we conducted a baseline evaluation of the number of neutrophils in peripheral blood and saliva and the ability of neutrophils to kill *C. albicans* in a laboratory in vitro assay (Table 1). The number of baseline peripheral (but not oral) neutrophils was higher in the subjects that later developed oral candidiasis. The causes for this difference were not clear, as a higher baseline number of neutrophils in the candidiasis-positive group was not related to the frequency of steroid premedication, prior to chemotherapy, a known inducer of neutrophilia [48] (Table 1). Despite differences in peripheral neutrophils at baseline, the number of oral neutrophils and their ability to kill *C. albicans* at this visit did not differ among oral candidiasis-positive and unaffected individuals. 

Next, we checked if baseline, oral, microbiome-related characteristics of subjects were associated with the development of oral candidiasis. The plaque index, a measurement of the amount of visible dental plaque was significantly different between oral candidiasis-positive and negative subjects with a higher baseline plaque index in subjects that later developed oral candidiasis (Table 1). We also measured the salivary bacterial and *Candida* load via qPCR assays and characterized the bacteriome and mycobiome via high throughput sequencing of the 16S rRNA gene and the ITS1 region, respectively. As seen in Table 1, baseline salivary bacterial and *Candida* load did not differ between oral-candidiasis-positive and oral-candidiasis-negative subjects. However, the bacteriome and mycobiome alpha-diversity was lower in subjects that later developed oral candidiasis, suggesting that certain oral microbiome communities are associated with a higher risk of developing the disease.

We found that bacteriome communities were highly diverse, contrary to mycobiome communities which were dominated by two main genera—*Candida* and *Malassezia*. The percentage of these genera in baseline saliva samples was different between subjects that developed oral candidiasis and those that remained candidiasis-free, with all subjects that developed the disease showing mycobiome communities dominated by *Candida* (83.2–99.9% of all fungal reads), and near absence or low proportions of *Malassezia* (Table 1).

### 3.2. Multivariate Analysis of Baseline Demographic, Medical, Oral, Neutrophil, and Microbiome Characteristics That Discriminated Between Oral-Candidiasis-Positive and Oral-Candidiasis-Negative Subjects 

**Figure 1:**
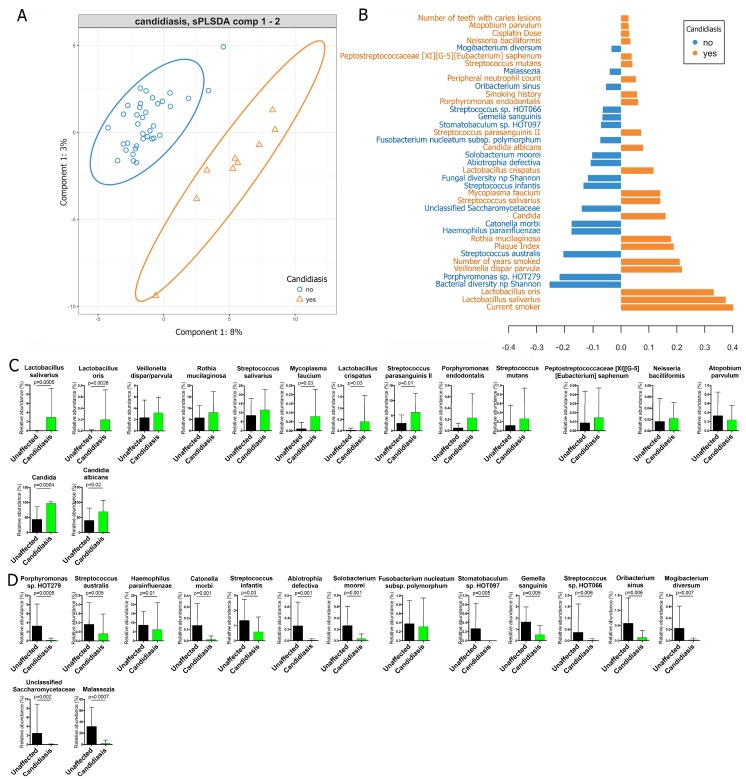
Multivariate sparse partial least square discriminant analysis (sPLS-DA) to identify baseline variables that are able to differentiate subjects that later developed oral candidiasis during chemotherapy (*n* = 9) from those that remained candidiasis-free (*n* = 36). (**A**) Separation of subjects that tested positive and negative for oral candidiasis, according to the sPLS-DA model. (**B**) Variables with their loadings contributing to component 1, which best separated subjects based on an oral candidiasis diagnosis. All baseline variables measured were taken into account in the analysis, including demographic, medical, oral-health, and neutrophil characteristics, bacterial and *Candida* burdens, microbiome diversity, and the proportions of microbiome taxa present in at least 10% of subjects. (**C**) The baseline proportions of bacterial (1st row) and fungal (2nd row) taxa, identified in the sPLS-DA model as ‘increased’ in subjects that later developed oral candidiasis. (**D**) The baseline proportions of bacterial (1st row) and fungal (2nd row) taxa, identified by the sPLS-DA model as ‘increased’ in the unaffected subjects. *p* values (unadjusted) shown in **C** and **D** are those that were significant after comparing the proportions via Mann–Whitney rank tests.

Next, we used sparse partial least square discriminant analysis (sPLS-DA), which enabled the selection of the most predictive or discriminative features in a dataset [44], for a multivariate analysis of the baseline characteristics that could best separate subjects who developed oral candidiasis, from the unaffected individuals. The model presented in Figure 1A, which had a cross-validated the overall classification error rate of ~15%, separated the subjects into two clusters, according to the oral candidiasis diagnosis. Figure 1B shows the variables driving the separation along component 1, the main component discriminating candidiasis-positive and candidiasis-negative subjects. Being a current smoker and having a high Plaque Index were the baseline clinical characteristics contributing to a shift towards the positive side of component 1 and were, therefore, associated with the development of oral candidiasis. Alongside these variables, higher baseline proportions of several species of *Lactobacillus*, *Streptococcus parasanguinis* II, *Veillonella dispar/parvula*, and *Streptococcus mutans*, among others, were also associated with oral candidiasis (Figure 1B). The baseline levels of *Candida*, in particular *C. albicans*, were the only mycobiome features associated with oral candidiasis (Figure 1B). However, it is worth noting that the effect of some bacteriome members was greater than the effect of the baseline proportions of *Candida*. In contrast, having baseline salivary bacteriome and mycobiome communities of higher diversity, higher baseline levels of bacteria such as *Porphyromonas* sp. HOT 279, *Streptococcus australis*, *Haemophilus parainfluenzae*, and *Catonella morbi*, among others, and higher baseline proportions of fungi such as unclassified *Saccharomycetaceae* and *Malassezia*, were negatively associated with the development of oral candidiasis (Figure 1B). Figure 1C,D show the baseline proportions of the bacterial and fungal taxa identified in the sPLS-DA analysis as discriminating between candidiasis-positive and unaffected individuals. In conclusion, although only twenty percent of the subjects were positive for oral candidiasis, it was still possible to develop a multivariate model of baseline factors associated with the infection. This model pointed to smoking, amount of visible plaque, low bacteriome and mycobiome diversity, and specific bacteriome and mycobiome components as risk factors for the development of oral candidiasis during chemotherapy. 

### 3.3. Characterization of Clinical Factors Associated With the Longitudinal Progression of Oral Candidiasis during Chemotherapy

**Figure 2:**
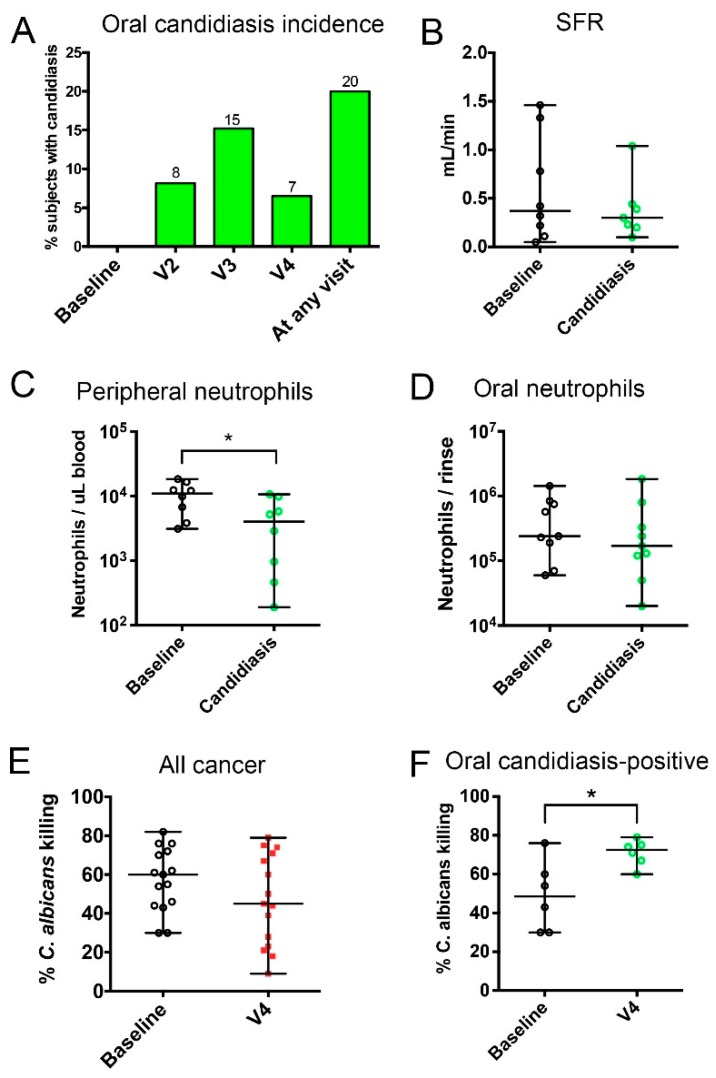
Incidence of oral candidiasis during one chemotherapy cycle and associated changes in salivary flow and neutrophil surveillance. (**A**) Oral candidiasis incidence. (**B**–**D**) Changes in salivary flow rate, peripheral neutrophil counts, and oral neutrophil counts between baseline and the first visit with candidiasis. (**E**,**F**) Changes between baseline and V4 in the ability of peripheral neutrophils to kill *C. albicans* in all cancer subjects and in subjects that tested positive for oral candidiasis, during the cycle. * indicates a *p* value < 0.05, when comparing subject-matched data via Wilcoxon Rank tests.

Next, we conducted an analysis of variables evaluated longitudinally by assessing their covariation with the development of clinical signs of oral candidiasis. As seen in Figure 2A, the highest number of subjects affected by oral candidiasis was seen at V3 (9 ± 2 days after chemotherapy). Table 2 shows the medications that the subjects received during the chemotherapy cycle. There was no relationship between receiving specific chemotherapeutic drugs or combination regimens and developing oral candidiasis. Importantly, receiving single or multi-dose systemic antibiotics was not associated with the development of candidiasis. Additionally, there was no association of any type of concomitant medication, including steroids, antibiotics or proton pump inhibitors, with oral candidiasis. As expected, receiving an antifungal, during chemotherapy, had a positive relationship with an oral candidiasis diagnosis.

To evaluate which variables—measured at multiple time points—correlated with the development of oral candidiasis, we modelled change over four visits with linear or quadratic polynomial contrasts. These contrasts represent the shape of the curve that each variable followed, in each subject, during the cycle. All subjects were included in this analysis and we used these contrasts to evaluate the correlation between curves followed by oral candidiasis and other metadata. Results from this analysis are shown in Table 3. A negative quadratic change in oral candidiasis (representing a down, up, down curve, which are subjects in which candidiasis was resolved by V4) correlated with a positive linear change in oral mucositis. This suggests that oral mucositis (erythema or ulcerations) occurred concomitantly, with or after a diagnosis of oral candidiasis, in patients that developed both conditions. With respect to changes in saliva availability, there was no difference in SFR at the visit with oral candidiasis, compared to baseline (Figure 2B). However, when we modelled the change in SFR in relation to oral candidiasis (Table 3), there was a correlation between having a decreased SFR in the middle of the cycle (positive quadratic curve—up, down, and up) and a linear increase in oral candidiasis, which suggested that decreased SFR preceded candidiasis clinical signs. 

We also evaluated the relationship of blood and salivary neutrophil counts with the incidence of oral candidiasis. A decrease in the peripheral neutrophil counts compared to the baseline, in the same individuals, was seen at the visit when subjects presented with candidiasis (Figure 2C), and as seen in Table 3, the negative quadratic (down, up, and down) or positive linear progression of oral candidiasis, correlated with decreased peripheral neutrophils counts, in the middle of the cycle (positive quadratic curve). It should be noted that peripheral neutrophils followed this pattern of longitudinal variation in most subjects, decreasing by V3 and rebounding by V4 [27]. In contrast, oral neutrophils did not decrease during oral candidiasis (Figure 2D) and there was no correlation between the longitudinal change in oral neutrophil counts and candidiasis (Table 3). These findings suggest that despite decreased peripheral neutrophils as a consequence of chemotherapy, these cells continued to infiltrate the oral tissues, as oral candidiasis occurred. 

We additionally evaluated the peripheral neutrophil function by a *C. albicans* killing assay. There was no change in function in the cancer group as a whole, during chemotherapy (Figure 2E). However, by V4 there was an increase in the killing capacity of neutrophils in subjects that experienced oral candidiasis (Figure 2F). Indeed, change in killing capacity correlated with the quadratic negative change (down, up, and down) in oral candidiasis (Table 3) and with increased oral *Candida* load at V3 (Spearman Rank test, *r* = 0.577, *p* = 0.049). These data suggest that *Candida* oral overgrowth sensitized the peripheral neutrophils. No other variable, including the intake of the granulocyte colony-stimulating factor analog pegfilgrastim, could explain this increase in function. Taken together, these data support a relationship between oral *Candida* colonization levels and functional priming of neutrophils, against the fungus.

### 3.4. Fungal and Bacterial Microbiome Changes in Chemotherapy-Associated Oral Candidiasis

**Figure 3:**
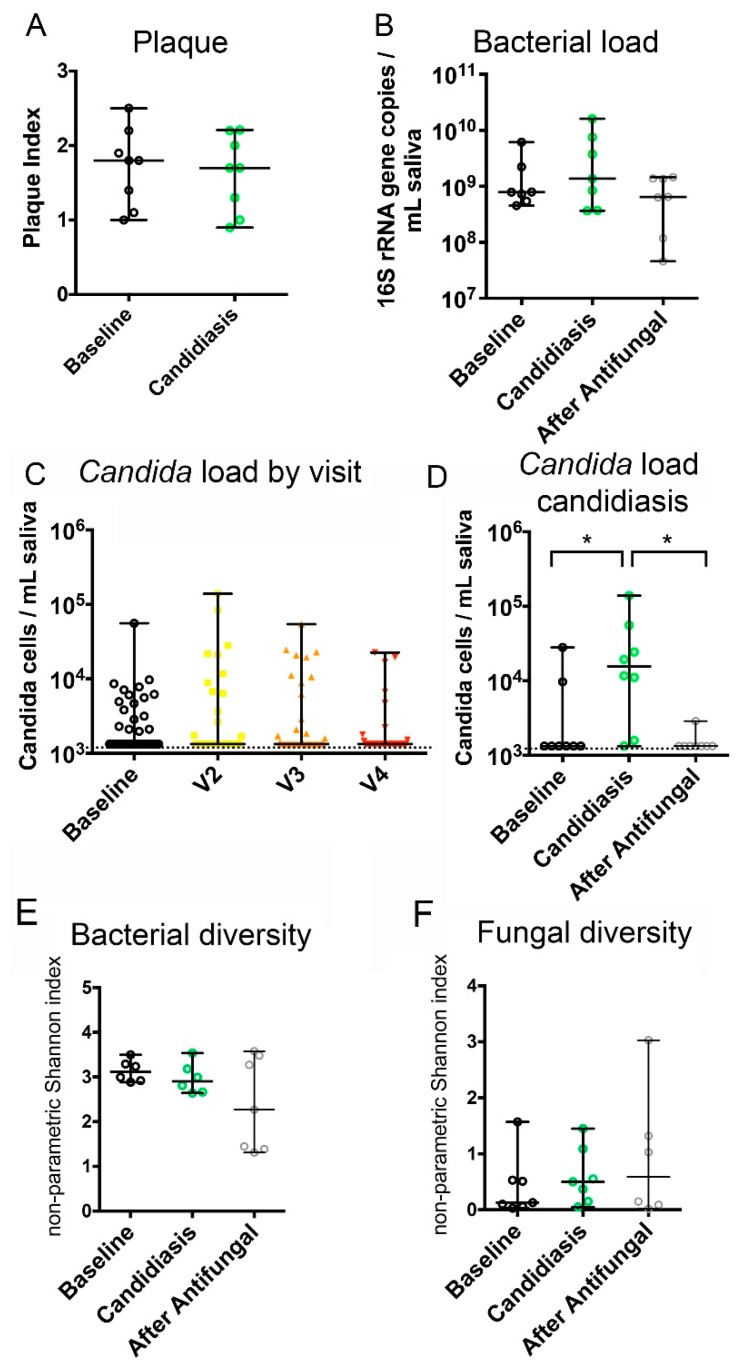
Changes in the bacterial and fungal burdens and community diversity during the development of oral candidiasis. (**A**) Changes in Plaque Index between baseline and the first visit with candidiasis. (**B**) Changes in salivary bacterial load as subjects developed oral candidiasis and after antifungal treatment. (**C**) Changes in salivary *Candida* load in all subjects, during the chemotherapy cycle. (**D**–**F**) Changes in *Candida* load, bacteriome diversity, and mycobiome diversity, as subjects developed oral candidiasis and after antifungal treatment. *indicates a *p* value < 0.05, when comparing subject-matched data via Wilcoxon Rank tests.

We evaluated if oral candidiasis was associated with longitudinal changes in salivary fungal and bacterial loads. As seen in Figure 3A, the Plaque Index did not differ between baseline and the visit when subjects presented with oral candidiasis. The salivary bacterial load showed a slight but not statistically significant increase from baseline, in subjects with oral candidiasis (Figure 3B) and there was no significant correlation between change over time in the bacterial load and the clinical development of oral candidiasis (Table 3). Our cancer cohort, as a whole, did not experience significant changes in salivary *Candida* biomass, over the course of the chemotherapy (Figure 3C). As expected, there was a statistically significant increase in the *Candida* load, as oral candidiasis signs developed and a decrease after the antifungal therapy (Figure 3D). Indeed, Table 3 shows that the negative quadratic change (down, up, and down curve) in the *Candida* load, correlated with either the negative quadratic or the positive linear change, in the presence of oral candidiasis clinical signs. 

The diversity of salivary bacterial and fungal communities did not change significantly during the course of oral candidiasis (Figure 3E,F) and there was no correlation between the change patterns in these variables and oral candidiasis development (Table 3). We also found no association between the development of oral candidiasis and the intake of medications during the cycle that could alter the oral microbiome or its relationship with the mucosa, including antibiotics, steroids, and acid inhibitors (Table 3). 

**Figure 4:**
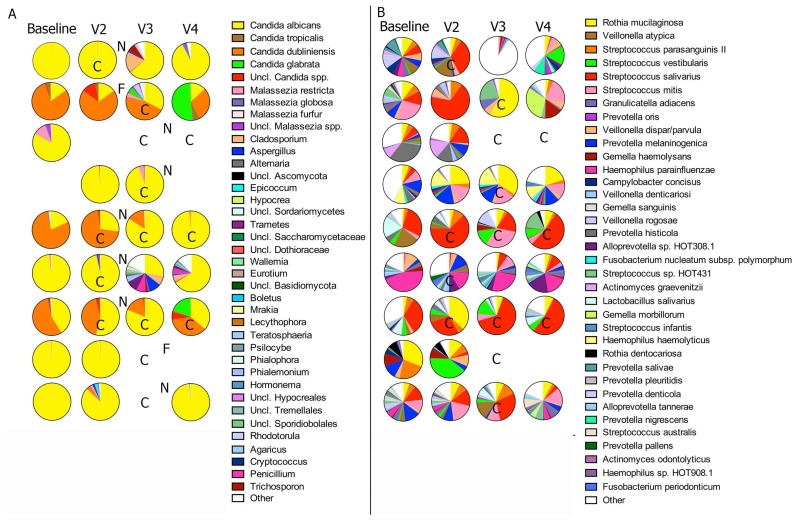
Longitudinal characterization of the oral microbiome during oral candidiasis development and after antifungal use. Salivary mycobiome (**A**) and bacteriome (**B**) composition of individual samples, in subjects that developed oral candidiasis during chemotherapy. The pie charts show the relative proportions of fungal taxa or bacterial species. C = oral candidiasis; N = Nystatin intake; F = Fluconazole intake. Only taxa present at a relative abundance of at least 1% (for fungi) and 5% (for bacteria) in one sample are depicted individually in the pie charts. “Other” indicates the aggregated counts of taxa not individually depicted.

Figure 4 shows the mycobiome and bacteriome composition at all visits during the cycle, in subjects that developed oral candidiasis. At almost all time-points, irrespective of oral candidiasis clinical signs or antifungal intake, mycobiome communities were dominated by *Candida*, with *C. albicans* and *Candida dubliniensis* as the most abundant species. Two subjects also showed an increase in the proportions of *Candida glabrata* by the end of the cycle (Figure 4A). In an sPLS regression analysis, to assess correlations between the longitudinal changes in the abundance of microbial taxa during the cycle and the presence of clinical signs of oral candidiasis, no fungal or bacterial taxon appeared as significantly correlated with candidiasis. As seen in Figure 4B, no evident patterns of change in the bacteriome species proportions were seen, as clinical signs of oral candidiasis developed. Taken together, these results showed that candidiasis development was not associated with longitudinal microbiome structure shifts but was the result of an increased *Candida* load, compared to the baseline levels.

## 4. Discussion

One of the common secondary effects of cancer chemotherapy treatment is oropharyngeal candidiasis, a common condition affecting immunocompromised populations [3,49,50,51]. Older studies in solid organ tumor cohorts used culture and biochemical phenotyping methods and identified *C. albicans*, followed by *C. tropicalis* and *C. glabrata* as the most frequent species associated with this infection (reviewed in [3]). Using next generation sequencing methods, we confirmed that *C. albicans* is the main species associated with oral candidiasis, in the chemotherapy setting, but also identified *C. dubliniensis* as the second most abundant species, in a subset of subjects who developed oral candidiasis. Due to its phenotypic resemblance to *C. albicans*, it is possible that previous studies have misidentified *C. dubliniensis* as *C. albicans* and underestimated its association with oral infection in this population [52]. This might have clinical implications as oral *C. dubliniensis* isolates from patients with hematologic malignancies have been reported to develop resistance to fluconazole, a mainstream prophylactic antifungal used in chemotherapy recipients [4]. Importantly, this is the first time a high throughput amplicon sequencing approach was used, to longitudinally analyze the mycobiome in subjects who developed oral candidiasis during cancer chemotherapy. Our analyses showed that the infection was not due to the acquisition of a new fungal species or structural community changes, but due to the increased load of species that were part of the commensal communities pre-treatment. We found that *Candida* was dominant in the mycobiome of all subjects, prior to developing oral candidiasis, and this mycobiome composition was preserved as clinical signs appeared, with candidiasis mainly characterized by increased *Candida* load when clinical signs were present. This confirms the findings of a longitudinal study that used molecular fingerprinting approaches, to follow patients with HIV-related immunosuppression who developed oral candidiasis [53]. Importantly, antifungals were able to decrease the *Candida* load but did not significantly alter the mycobiome composition, with *Candida* still remaining the most abundant fungal commensal, after treatment. Antifungals, therefore, did not modify the future risk of oral candidiasis, as conferred by a *Candida*-dominated baseline community. 

ITS-1-based analyses in this chemotherapy cohort suggest that a *Malassezia*-dominated baseline salivary mycobiome is associated with low *Candida* burdens and protection from oral infection. Our group was the first to identify *Malassezia* as a prominent oral mycobiome member in healthy individuals, with relative abundance ranging from 13% to 96% [12]. It is possible that the *Candida* and *Malassezia* species have an antagonistic relationship in the oral cavity that is driven by ecologic competition for nutritional resources. Alternatively, it is possible that *Malassezia* limits *Candida* growth by secreting mannosidases and other glycosyl hydrolases, which damage the *Candida* cell wall [54]. Despite the fact that *Candida* and *Malassezia* are dominant eukaryotic residents of the human oral cavity, nothing is known with respect to their interactions and their potential role in preventing infection.

Prior to our study, at least four other studies showed that chemotherapy alone almost doubles oral *Candida* carriage rates, compared to pre-treatment in cancer patients (reviewed by [3]). In our study, when the entire chemotherapy population was analyzed longitudinally, there were no significant differences in oral *Candida* burdens between baseline and subsequent post-treatment visits. Differences in methodology (culture/biochemical identification in prior studies versus molecular quantification in this study, timing and method of sampling, type of chemotherapy agents, etc.) might have contributed to this discrepancy. However, in mice that were orally inoculated with *C. albicans* while receiving 5-fluorouracil, we showed that *Candida* burdens increased in a time-dependent fashion and peaked when mice developed oral pseudomembranous candidiasis [26]. In this chemotherapy patient cohort, candidiasis occurred in patients whose oral mycobiome was dominated by *C. albicans* pre-treatment, whereas infection was temporally associated with a further increase in *Candida* burdens, consistent with our mouse model. It should also be noted that our ITS-1-based analyses only discriminated different species. Strain variability might also be important in determining susceptibility to oral candidiasis and might explain why some individuals with high *Candida* carriage remained asymptomatic during chemotherapy (Table 1).

A chemotherapy-induced increase in oral commensal bacterial burdens, particularly aciduric bacteria, such as lactobacilli and streptococci, has been reported in a chemotherapy-treated human breast cancer cohort [55]. This is in agreement with our mouse chemotherapy model where a time-dependent increase in oral bacterial counts takes place in response to 5-FU treatment, which is further accentuated by *C. albicans* infection. Similar to our mouse model, in this human cohort, oral candidiasis was associated with a small, albeit not statistically significant, increase in oral bacterial burdens. Interestingly, in this patient cohort, subjects who were at an increased risk for oral candidiasis had a lower bacterial diversity and they were more abundantly colonized by aciduric bacteria including certain *Lactobacillus* and *Streptococcus* species, prior to developing the infection, suggesting that these bacteria might be risk factors underlying susceptibility. In particular, *L. salivarius*, *L. oris*, *L. crispatus*, and *S. parasanguinis*, a member of the Mitis group of oral streptococci, were significantly more abundant in patients who went on to develop oral candidiasis. Our findings suggest that when sufficiently abundant, these species might place this population at an increased risk for subsequent infection. There is mounting experimental and clinical evidence (observed by our group and others) of a pathogenic synergy or at least of a mutualistic relationship between *C. albicans* and the Mitis group streptococci [25,56,57,58]. However, this synergy is likely to be highly species-specific within members of this streptococcal group, since another Mitis group member (*S. infantis*) was less abundant in patients who later developed oral candidiasis. *Lactobacillus* species have traditionally been associated with protection from vaginal candidiasis, and several molecular mechanisms of antagonism with *C. albicans* have been discovered (reviewed in [59]). With the exception of *L. crispatus*, there is little overlap between the member species of the oral and vaginal mucosa, under physiological conditions [10,60]. Thus, differences in metabolic activities among *Lactobacillus* species abundantly colonizing the two sites might be responsible for their opposite associations with oral and vaginal candidiasis.

In our chemotherapy cohort, oral mucositis occurred at the same time or shortly after oral candidiasis, which suggests that mucositis does not increase risk of oral candidiasis. In agreement with this, previous longitudinal studies in intensive chemotherapy cohorts have found no positive risk associations between the two oral comorbidities [4,61]. However, the finding of both conditions occurring simultaneously might have serious clinical implications, since mucositis compromises the mucosal barrier and facilitates fungemia [62].

We found a correlation, however, between systemic myelosuppression, as evidenced by the peripheral neutrophil counts and oral candidiasis. Despite the decreased peripheral counts, neutrophil function (as assessed by an ex-vivo *C. albicans* killing assay) increased by V4, in subjects that developed oral candidiasis at any point during the cycle or had a higher *Candida* load at V3. This might suggest that, at least a subset of these cells evolved to an adaptive state that maintained a higher level of activation, in order to protect the host. This adaptive state, known as trained innate immunity, is based on epigenetic reprogramming and has been shown to be induced by *C. albicans* or fungal β-glucans, in innate immune cells (reviewed in [63]). Furthermore, we did not observe a deficiency in oral neutrophil numbers, which suggests that neutrophil migration into the oral tissues was still taking place. In fact, decreased oral neutrophil availability did not correlate with oral candidiasis. Instead, other clinical factors known to disturb local protective mechanisms were associated with oral candidiasis. For example, a decreased salivary flow appeared to precede oral candidiasis. Other studies have linked impaired salivary flow to increased *Candida* carriage [64] and oral candidiasis [65], possibly due to a lower oral availability of *Candida*-controlling antimicrobial proteins. Smoking was the second factor found to be associated with a dysregulation of local defenses and to confer an oral candidiasis risk. The relationship between smoking and oral candidiasis is controversial, with some studies supporting an association, while others not finding a link [65,66]. Cigarette smoke has been shown to increase inflammatory responses of the airway epithelium [67], and smoking is associated with a dysregulated expression of human beta defensins in oral mucosa [68]. It is possible that smoking, in the setting of chemotherapy, becomes even more important, potentially interacting with cytotoxic drugs to dysregulate mucosal homeostasis, facilitating *Candida* overgrowth and tissue damage.

This study contributes to a better understanding of the interplay between the myelosuppressive effects of chemotherapy, the oral microbiome, and the development of oral candidiasis. We outline novel relationships between *Candida* and bacterial microbiome members, which might be significant in the pathophysiology of oral candidiasis in humans. Moreover, in an integrated analysis of baseline clinical and microbiome characteristics associated with development of oral candidiasis, we identified smoking, amount of visible dental plaque, low bacteriome and mycobiome alpha-diversity, and relative abundance of specific bacterial and fungal taxa as baseline factors associated with a risk of oral candidiasis. We presented evidence that a baseline *Candida*-dominated mycobiome, together with a number of aciduric bacteria, such as oral streptococci and lactobacilli, might be important determinants of susceptibility to this infection. Our studies represent a significant first step toward the development of clinical and oral microbiome risk profiles, as a tool to identify immunocompromised patients that are at a higher risk of oral candidiasis. Such tools will be instrumental in delivering well-targeted, patient-specific, preventive antimicrobial approaches, prior to the onset of oral infection and potential systemic dissemination.

## Figures and Tables

**Table 1:** Baseline characteristics of the study subjects according to the oral candidiasis diagnosis during the chemotherapy cycle. All data represent information collected or measurements taken at baseline, prior to the chemotherapy cycle.

Variable	No Candidiasis n = 36	Oral Candidiasis n = 9	Statistic
Age	57.9 ± 13.6	56.6 ± 10.8	0.788 *
Gender (% male)	50.0	77.8	0.260 ^@^
Race (% white)	86.1	100.0	0.566 ^@^
Ethnicity (% hispanic)	5.6	0.0	1.000 ^@^
Squamous-cell carcinoma (% yes)	36.1	66.7	0.137 ^@^
Breast cancer (% yes)	33.3	11.1	0.248 ^@^
Adenocarcinoma (% yes)	25.0	11.1	0.659 ^@^
Other cancer (% yes)	5.6	11.1	0.497 ^@^
**Smoking history (% yes)**	**58.3**	**100.0**	**0.020 ^@^**
Former smoker (% yes)	55.6	33.3	0.284 ^@^
**Current smoker (% yes)**	**2.8**	**66.7**	**0.00007 ^@^**
Proton pump inhibitor use (% yes)	27.8	11.1	0.416 ^@^
Inhaler steroid use (% yes)	2.8	11.1	0.364 ^@^
Steroid premedication prior to V1 (% yes)	16.7	22.2	0.651 ^@^
Number of teeth	26 (0–32)	21 (0–28)	0.190 ^&^
Prosthetic teeth (% yes)	77.8	77.8	1.000 ^@^
Number of teeth replaced by prosthesis	2 (0–25)	2 (0–32)	0.967 ^&^
Removable prosthesis (% yes)	13.9	11.1	1.000 ^@^
Visible caries lesions (% yes)	30.6	44.4	0.454 ^@^
Number of teeth with caries lesions	0.81 ± 1.5	2.56 ± 3.7	0.200 *
**Plaque index**	**0.9 (0.0–2.1)**	**1.8 (1.0–2.5)**	**0.001 ^&^**
Periodontal status (CPITN, %) Healthy Bleeding Calculus or defective restorations Periodontal pocket between 4 and 5 mm Periodontal pocket > 6 mm Edentulous	5.6 2.8 41.7 33.3 13.9 2.8	0.0 0.0 22.2 66.7 0 11.1	0.322 ^#^
Salivary flow rate (mL/min)	0.39 (0.06–0.93)	0.42 (0.05–1.46)	0.625 ^&^
**Peripheral absolute neutrophil count** **(× 1000/mm^3^ blood)**	**5.4 (2.2–16.1)**	**12.1 (3.1–18.4)**	**0.039 ^&^**
Oral neutrophil count (**×** 10,000/rinse)	44 (6–473)	24 (6–143)	0.309 ^&^
*C. albicans* killing by peripheral neutrophils (% killed *C. albicans*) n = 14 subjects^	62.2 (30–82)	53.5 (30–76)	0.240 ^&^
Bacterial load (16S rRNA copies/mL saliva)	4.9E+09 ± 4.8E+09	2.0E+09 ± 2.1E+09	0.104 *
*Candida* load (*Candida* cells/mL saliva)	2.5E+03 ± 2.1E+03	5.2E+03 ± 9.0E+03	0.399 *
**Salivary bacterial diversity (16S rRNA gene based—np Shannon Index)**	**3.2** **± 0.31**	**2.6** **± 0.40**	0.0001 *
**Salivary fungal diversity (ITS1 based—np Shannon Index)**	**0.99** **± 0.81**	**0.14** **± 0.17**	0.00001 *
**Percentage *Candida***	**27.7 (0.0–99.6)**	**98.7 (83.2–99.9)**	**0.0005 ^&^**
**Percentage *Malassezia***	**16.0 (0.0–98.4)**	**0.1 (0–15.6)**	**0.0007 ^&^**

The distribution of continuous data was tested for normality, using measures of Skewness and Kurtosis and the Shapiro–Wilk test in SPSS. For the normally-distributed continuous variables, mean ± standard deviation is shown. For the non-normally distributed continuous variables, median is shown, with the range in parentheses. Statistical tests for continuous data were applied according to the data distribution. For non-continuous variables, the data shown represent the percentage of subjects that tested positive. ^ Of the 14 subjects included in the *C. albicans* killing assays, 9 did not present oral candidiasis during the cycle and 5 developed oral candidiasis. ^#^ chi-square; ^@^ Fisher’s exact test; ^&^ Mann–Whitney U test; * Independent sample t-test. Variables that differed between candidiasis-positive subjects and unaffected individuals are shown in bold.

**Table 2:** Medications received during the cycle according to candidiasis diagnosis.

	No Candidiasis *n* = 36	Oral Candidiasis *n* = 9	Statistic
5-FU	66.7	77.8	0.698 ^@^
Doxorubicin	33.3	22.2	0.698 ^@^
Docetaxel	38.9	66.7	0.157 ^@^
Cyclophosphamide	33.3	11.1	0.249 ^@^
Cisplatin	27.8	66.7	0.050 ^@^
Carboplatin	16.7	11.1	1.000 ^@^
Oxaliplatin	13.9	11.1	1.000 ^@^
Leucovorin	16.7	11.1	1.000 ^@^
Herceptin	2.8	11.1	0.364 ^@^
Mitomycin	2.8	0.0	1.000 ^@^
Bevacizumab	5.6	0.0	1.000 ^@^
Irinotecan	2.8	0.0	1.000 ^@^
Vinblastine	0.0	11.1	0.200 ^@^
5FU-docetaxel-cisplatin	25.0	55.6	0.111 ^@^
5FU-docetaxel-carboplatin	13.9	11.1	1.000 ^@^
5FU-oxaliplatin-leucovorin	8.3	0.0	1.000 ^@^
5FU	5.6	0.0	1.000 ^@^
5FU-mitomycin	2.8	0.0	1.000 ^@^
5FU-oxaliplatin-leucovorin-herceptin	2.8	11.1	0.364 ^@^
5FU-cisplatin-carboplatin	2.8	0.0	1.000 ^@^
5-FU-irinotecan-Bevacizumab-leucovorin	2.8	0.0	1.000 ^@^
5FU-oxaliplatin-leucovorin-Bevacizumab	2.8	0.0	1.000 ^@^
Doxorubicin-cyclophosphamide	33.3	11.1	0.249 ^@^
Doxorubicin-cisplatin-vinblastine	0.0	11.1	0.200 ^@^
Steroid during cycle	80.6	55.6	0.190 ^@^
Pegfilgrastim	72.2	88.9	0.416 ^@^
Proton pump inhibitors	66.7	77.8	0.698 ^@^
Any antibiotic	38.9	66.7	0.157 ^@^
Single dose prophylactic	22.2	33.3	0.666 ^@^
Multi-dose antibiotic	19.4	33.3	0.393 ^@^
**Any antifungal**	**2.8**	**100.0**	**0.0000001**
**Nystatin**	**0.0**	**77.8**	**0.000001**
Fluconazole	2.8	22.2	0.097

Data represent the percentage of subjects that received the medication. ^@^ Fisher’s exact test. Variables that differed between candidiasis positive subjects and unaffected individuals are shown in bold.

**Table 3:** Correlation between change in oral candidiasis signs during the cycle and the change in clinical and microbiome characteristics measured longitudinally. All 45 subjects were included in these analyses.

	Significance Test vs. Zero (*p* Value)	Clinical Oral Candidiasis (Q)	Clinical Oral Candidiasis (L)
Clinical oral candidiasis(Q)	0.004	-	−0.380(0.008)
Clinical oral candidiasis (L)	0.057	−0.380(0.007)	-
Oral mucositis (Q)	<0.001	0.094(0.876)	0.177(0.233)
Oral mucositis (L)	<0.001	−0.389(0.008)	0.115(0.432)
Salivary flow rate (Q)	0.742	−0.278(0.058)	0.401(0.005)
Salivary flow rate (L)	0.002	−0.081(0.587)	−0.174(0.238)
Oral neutrophil count (Q)	0.398	−0.060(0.668)	0.057(0.703)
Oral neutrophil count (L)	0.623	−0.269(0.068)	−0.127(0.396)
Peripheral neutrophil count (Q)	<0.001	−0.483(0.001)	0.522(<0.001)
Peripheral neutrophil count (L)	0.360	−0.311(0.033)	0.521(<0.001)
*C. albicans* killing by peripheral neutrophils (L) ^	0.200	−0.614(0.015)	0.336(0.220)
Plaque Index (Q)	0.767	−0.148(0.331)	0.124(0.418)
Plaque Index (L)	0.940	−0.058(0.703)	−0.166(0.274)
Salivary bacterial load (Q)	0.492	0.075(0.647)	−0.215(0.182)
Salivary bacterial load (L)	0.031	−0.035(0.825)	0.031(0.842)
Salivary *Candida* load (Q)	0.013	0.335(0.034)	−0.436(0.005)
Salivary *Candida* load (L)	0.405	0.297(0.053)	−0.038(0.808)
Salivary bacterial diversity (Q)	0.051	−0.193(0.220)	0.100(0.527)
Salivary bacterial diversity (L)	0.009	−0.175(0.250)	−0.067(0.656)
Salivary fungal diversity (Q)	0.514	0.190(0.282)	0.109(0.539)
Salivary fungal diversity (L)	0.435	−0.285(0.064)	−0.097(0.529)
Antibiotics (any) (Q)	0.183	0.104(0.498)	−0.084(0.584)
Antibiotics (any) (L)	0.026	−0.013(0.935)	0.167(0.273)
Multi-dose antibiotic (Q)	0.533	0.163(0.283)	−0.265(0.079)
Multi-dose antibiotic (L)	0.004	0.071(0.641)	0.094(0.539)
Single-dose antibiotic (Q)	0.019	−0.038(0.806)	0.074(0.627)
Single-dose antibiotic (L)	0.710	−0.003(0.985)	0.056(0.717)
Steroids (Q)	<0.001	−0.252(0.095)	0.101(0.510)
Steroids (L)	<0.001	−0.118(0.439)	−0.047(0.761)
Acid Inhibitors (Q)	0.001	0.201(0.186)	−0.220(0.147)
Acid inhibitors (L)	0.685	0.051(0.741)	0.130(0.395)

As described in the Methods section, the longitudinal analysis of oral candidiasis development and the change in other variables measured at more than one time-point were evaluated using linear (L) or quadratic (Q) orthogonal polynomial contrasts. Data from each variable were transformed using either linear or quadratic four-level coefficients, and was aggregated by subject. For quadratic change, a positive value refers to a “U”-shaped curve (i.e., high to low to high) and negative indicates an “inverted-U”-shaped curve (i.e., low to high to low). For linear change, a positive indicates an upward change, while a negative value indicates a downward linear change from baseline. Values from each variable were evaluated to establish if the change differed from zero, via a *t*-test and were tested for their correlation with other variables, to evaluate significant covariation patterns. Data shown in the two columns on the right are the correlation coefficients (Spearman); *p* values are shown in parentheses. Significance thresholds for each outcome variable (in columns) were adjusted for multiple comparisons, via the FDR method. Values in red indicate correlations that were significant after FDR adjustment. Values in yellow indicate correlations with a *p* value < 0.05 but not significant after FDR adjustment. ^ *C. albicans* killing by peripheral neutrophils was only evaluated in a linear manner, as the data only included two time-points (baseline and V4).
